# Comparative efficacy of aspirin versus direct oral anticoagulants for venous thromboembolism prophylaxis following primary total hip arthroplasty or total knee arthroplasty: A systematic review and meta‐analysis of randomised controlled trials

**DOI:** 10.1002/jeo2.70010

**Published:** 2024-09-02

**Authors:** Fauzaan Ali Syed, Hamzah Amin, Biju Benjamin, Michiel Hendrix, Terence Savaridas

**Affiliations:** ^1^ Faculty of Health and Medicine, Lancaster University Medical School Lancaster University Lancaster UK; ^2^ Department of Orthopedic Surgery NHS Forth Valley Larbert Scotland

**Keywords:** aspirin, DOACs, THA, TKA, VTE prophylaxis

## Abstract

**Introduction:**

Venous thromboembolisms (VTEs), including deep vein thrombosis (DVT) and pulmonary embolisms (PE), are common after total knee (TKA) and hip arthroplasty (THA). Recent studies suggest that aspirin effectively prevents VTE following major orthopaedic surgery. This meta‐analysis compares randomised controlled trials (RCTs) evaluating aspirin versus direct oral anticoagulants (DOACs) for VTE prevention after primary THA and TKA.

**Methods:**

We included RCTs from 2017 to 2023 that looked at aspirin versus DOACs for VTE prophylaxis in primary THA and TKA. A search strategy was conducted which used Boolean operators and MESH terms. Primary outcomes included VTE rates, symptomatic, asymptomatic DVT and PE. Secondary outcomes were mortality and bleeding complications. Statistical analysis was performed using REVMAN software. An odds ratio with a 95% confidence interval was generated for the pooled studies. Heterogeneity was assessed using the *I* ^2^ variable, and publication bias was evaluated with a funnel plot.

**Results:**

Seven RCTs with 3967 patients were included for analysis. Rivaroxaban 10 mg OD was compared to varying doses of aspirin (81–300 mg). There were no significant differences between the groups in the incidence of VTE (OR: 1.21, 95% CI: 0.72–2.01), PE (OR: 1.01, 95% CI: 0.39–2.61), asymptomatic DVT (OR: 1.39, 95% CI: 0.64–3.00), suspected DVT (OR: 1.13, 95% CI: 0.49–2.61) and major bleeding (OR: 0.84, 95% CI: 0.55–1.27).

**Discussion:**

Aspirin is as effective as rivaroxaban for primary thromboprophylaxis post‐THA and TKA, without increased incidence of complications. Further research is needed to determine the optimal dosing regimen of aspirin and its long‐term efficacy in preventing VTE.

**Level of Evidence:**

Level I.

**Abbreviations**
CI: confidence Interval
DOAC: direct oral anticoagulant
DVT: deep vein thrombosis
MeSH: medical subject headings
NICE: National Institute for Health and Care Excellence
OR: odds ratio
PE: pulmonary embolism
PRISMA: preferred reporting items for systematic reviews and meta‐analyses
RCT: randomised controlled trial
THA: total hip arthroplasty
THR: total hip replacement
TKA: total knee arthroplasty
TKR: total knee replacement
VT: venous thrombosis
VTE: venous thromboembolism

## INTRODUCTION

Venous thromboembolism (VTE), including deep vein thrombosis (DVT) and pulmonary embolisms (PE), is a serious complication during or after hospitalisation for acute medical illness or surgery [7]. Elective primary total knee arthroplasty (TKA) and total hip arthroplasty (THA) both carry a high risk of VTE post‐operatively. Epidemiological studies from several studies globally suggest an incidence of VTE post‐TKA without prophylaxis of between 40% and 84%. Likewise having a THA also carries a significant risk of VTE (17%–57%) if no VTE prophylaxis is given [10, 24]. Furthermore, where patients do suffer a VTE after arthroplasty the risk of in‐hospital mortality is high at 7.1%, compared to 0.3% in those with no VTE [10, 24, 28].

The National Institute of Health and Care Excellence (NICE) in England recommends that all individuals undergoing elective arthroplasty should have VTE prophylaxis. Current NICE guidelines suggest the use of a low molecular weight heparin (LMWH) such as Enoxaparin or a direct oral anticoagulant (DOAC) such as rivaroxaban [21]. Rivaroxaban is often preferred to LMWH due to the convenience of oral tablet use, alongside safety and better efficacy [17, 26, 29]. Furthermore, aspirin in current guidance is an off‐label prescription that can be used as a sole prophylaxis agent in TKA but must be an adjunct for LMWH in THA in very limited clinical scenarios including patients with malignancy [21].

Aspirin is an affordable and widely available non‐steroidal anti‐inflammatory drug (NSAID). While it is well‐established for preventing cardiovascular events through its inhibition of platelet aggregation [30], its role in VTE prophylaxis post‐major orthopaedic surgery remains controversial [3, 6, 27]. Aspirin has been considered effective for preventing VTE after TKA and THA, though it is not routinely used [3, 6, 27]. Historically, aspirin has been supported by evidence for VTE prevention, however, recent advancements in peri‐operative care, including enhanced recovery protocols, have called this evidence into question [6]. These advances, combined with new anticoagulant medications (DOACs) being licensed in the 2010s, possibly accentuated the effects seen with DOACs compared to historical studies [6]. Consequently, despite potential efficacy, aspirin may be overshadowed by newer anticoagulants in current practice [1].

The widespread use of DOACs, largely due to the convenience of oral tablet use, is not without complications, with previous research suggesting that rivaroxaban is associated with wound leaks and bleeding complications [11, 22]. Furthermore, given that aspirin is widely and cheaply available this led us to consider whether aspirin could be used for VTE prophylaxis in the elective setting for primary TKA and THA. Haykal et al. conducted a meta‐analysis on this topic which included 13 RCTs involving 20,115 patients and compared aspirin to various anticoagulants (including warfarin, LMWH, DOACs and placebo) in TKA and THA both in the elective and acute setting [12]. Aspirin has reduced VTE when compared to placebo with a comparable safety profile. They also found aspirin to be as effective and safe as other anticoagulants (warfarin, LMWH and DOACs) with no significant difference in adverse events such as bleeding, rehospitalization, and mortality [12, 19]. Furthermore, in a subgroup analysis aspirin was found to be as effective as rivaroxaban for VTE prophylaxis albeit only three RCTs were included in the analysis [12].

However, other systematic reviews differ in their conclusions. Le et al. in their meta‐analysis found that aspirin and rivaroxaban had a similar effect in the prevention of VTE after TKA or THA; however, rivaroxaban had increased efficacy in preventing DVT, whilst aspirin resulted in less blood transfusions [11, 18]. Ultimately, the inclusion of lower‐quality evidence limits the conclusions we can make from their study. Consequently, there is a need for a systematic review that considers only the highest forms of clinical trial evidence (RCTs) and newer published studies to understand the role of aspirin as post‐arthroplasty VTE prophylaxis.

## AIMS AND OBJECTIVES

To conduct a systematic review and meta‐analysis of RCTs to assess whether aspirin can be used as a viable alternative to DOACs for VTE prophylaxis after elective hip and knee arthroplasty.

## METHODS

### Search strategy

**Figure 1:**
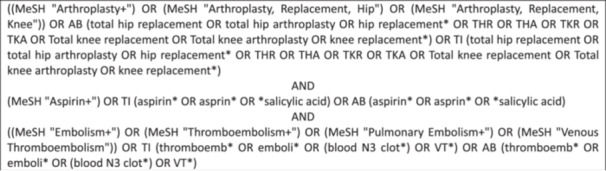
Sensitive search strategy.

A sensitive search strategy was developed using Boolean operators and MESH terms and formed the basis of our search as shown in Figure 1. A comprehensive literature search was conducted using the MedLine and EMBASE databases on 26 November 2023. Articles were included from 1 January 2017 to 26 November 2023. This allowed for 6 full years of results alongside the results from 2023. The rationale for this was to achieve a balance of having recent and relevant RCTs whilst having a breadth of information to draw conclusions from. Furthermore, we wanted to only include newer trials where VTE prophylaxis was given in conjunction with advances in better peri‐operative care to reflect current orthopaedic practice. Our strategy also enabled us to include studies not assessed in Le et al.'s meta‐analysis [18].

### Selection criteria

Studies were eligible for inclusion if they were in the English language and were randomised controlled trials (RCTs). The included RCTs compared the efficacy of aspirin to DOACs for venous thromboembolism (VTE) prophylaxis following primary elective THA or TKA in adult populations. Studies were excluded if they involved trauma patients due to concerns regarding immobilisation and a pro‐thrombotic state associated with a recent injury.

Primary outcomes included rates of VTE comprising symptomatic DVT (scanned due to clinical suspicion), asymptomatic DVT (found incidentally on scan) and pulmonary embolism (PE). Secondary outcomes were mortality and bleeding complications. Bleeding complications included major bleeding and all bleeding. Major bleeding was defined according to the definition used by Anderson et al., including intracranial or intramuscular bleeding (resulting in compartment syndrome), bleeding requiring blood transfusions or bleeding requiring reoperation [2]. All bleeding included all clinically relevant bleeding not limited to the major bleeding category above. Examples of non‐major bleeding included epistaxis and wound haematomas.

### Data extraction and quality assessment

The search strategy was independently conducted by two investigators. The titles were then exported into EndNote 21, where duplicates were automatically removed. The two investigators then separately reviewed the titles and abstracts of all papers to remove non‐related research. The investigators confirmed their selection, and any disagreements were resolved by the senior investigator. Once agreed upon, all selected studies were read in detail, and any additional papers not related or not meeting the eligibility criteria were removed. Throughout the process, a PRISMA flowchart was used for methodological transparency. Once a final list of papers was obtained, data were extracted into a Microsoft Excel document, including demographics and primary and secondary outcomes. The Cochrane risk of bias criteria was utilised to evaluate the quality of the included studies.

### Statistical analysis

Statistical analysis was conducted using Cochrane REVMAN software, which employs the Mantel–Haenszel method for pooling data. The effect was measured using a dichotomous variable and an odds ratio (OR) reported alongside a 95% confidence interval (95% CI). Heterogeneity was measured using the *I* ^2^ variable and was derived from the Cochrane Handbook; low: 0%–40%; moderate: 30%–60% and anything higher would constitute high levels of heterogeneity [9]. Due to the low volume of studies, we decided to accept a low‐moderate level of heterogeneity, with anything higher being grounds for a subgroup analysis to investigate the cause. A funnel plot was used to assess for the presence of publication bias. A symmetrical distribution around the combined effect size line was considered as low risk of publication bias while asymmetry around this line was considered as high risk of publication bias.

## RESULTS

### Search strategy: Results

**Figure 2:**
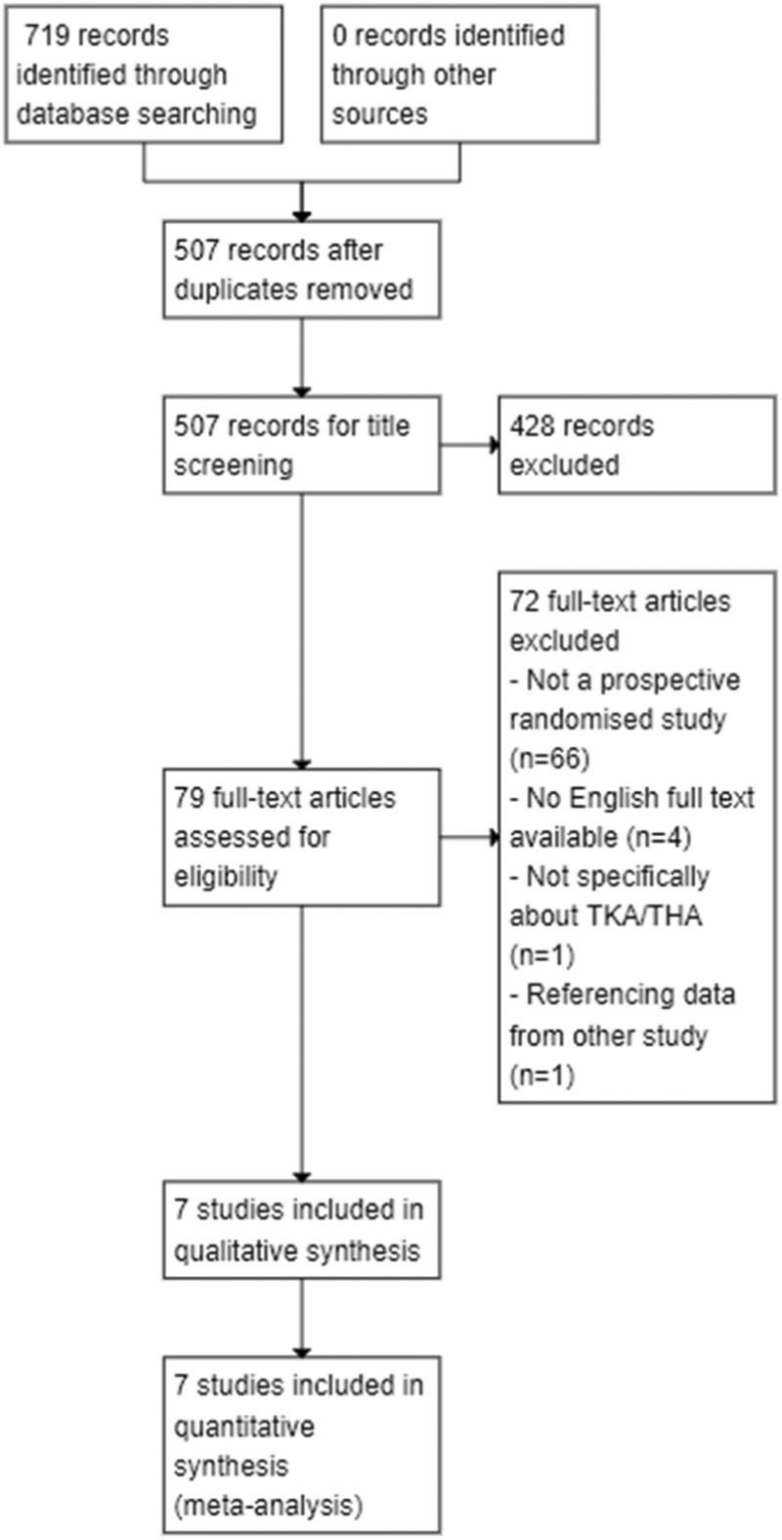
PRISMA flowchart demonstrating how the final number of studies was reached.

The search was conducted on 26 November 2023. Our search strategy yielded 719 results. After discarding duplicate entries, 507 results remained for analysis. After screening by two independent reviewers, seven studies were identified and included for analysis [2, 8, 13, 15, 25, 33, 34]. All studies that were selected for qualitative analyses had primary outcome data and thus were included in the quantitative analysis. A flow diagram explaining how the final numbers of RCTs was arrived at is demonstrated in Figure 2.

### Study characteristics

**Figure 3:**
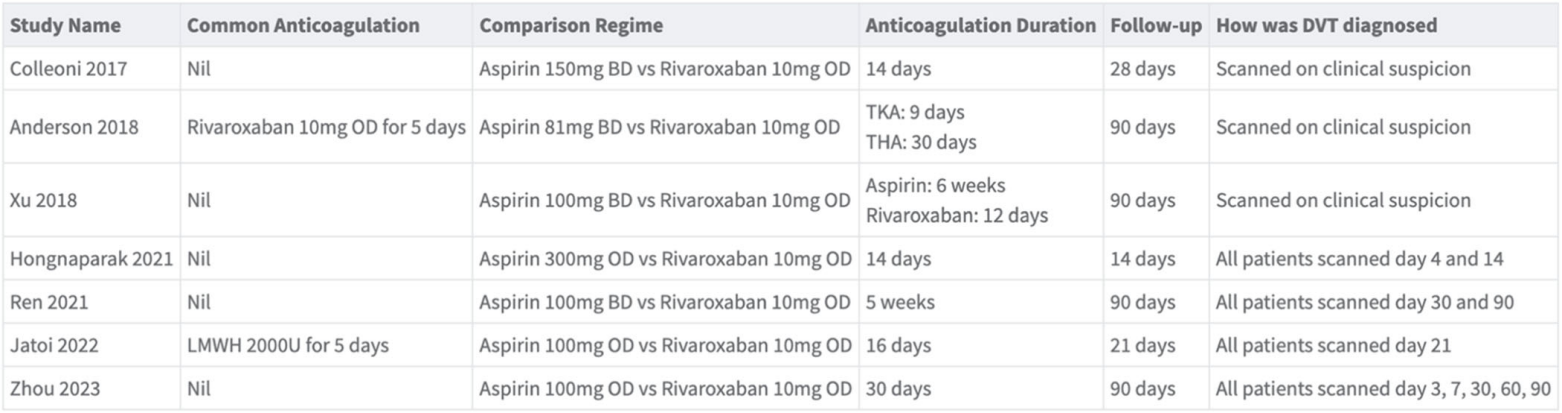
Table demonstrating study characteristics.

All seven studies included were RCTs. Each study compared an aspirin regime to a DOAC regime for post‐operative thromboprophylaxis and measured DVT incidence over a follow‐up time from 14 to 90 days. Some studies utilised a common anticoagulation regime for all patients before randomisation. The anticoagulation duration ranged from 14 days to 6 weeks. All studies used rivaroxaban as their DOAC of choice at a consistent dose of 10 mg once daily. Aspirin regimes differed by study, spanning 81 mg of aspirin daily to 300 mg of aspirin. Follow‐up time for the included study ranged from 14 to 90 days. The characteristics of each study is noted in Figure 3.

### Patient demographics

**Figure 4:**
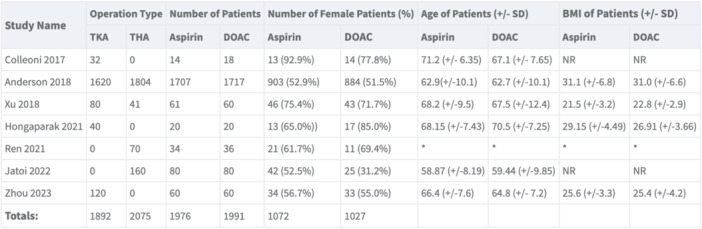
Table demonstrating patient demographics and operation type.

The 3967 patients were included for the analyses. The 1976 (49.8%) patients were in the aspirin group whereas 1991 (50.2%) were part of the rivaroxaban group. The 2099 participants were females (52.9%). The mean patient age of the aspirin group ranged from 58.9 to 71.2 years, whilst it was 59.4–70.5 years in the rivaroxaban group. BMI ranged from 25.6 to 31.1 kg/m^2 ^in the aspirin group compared to 25.4–31.0 in the rivaroxaban group. The patient demographics between the two groups were comparable. These data are summarised in Figure 4.

### Risk of bias

**Figure 5:**
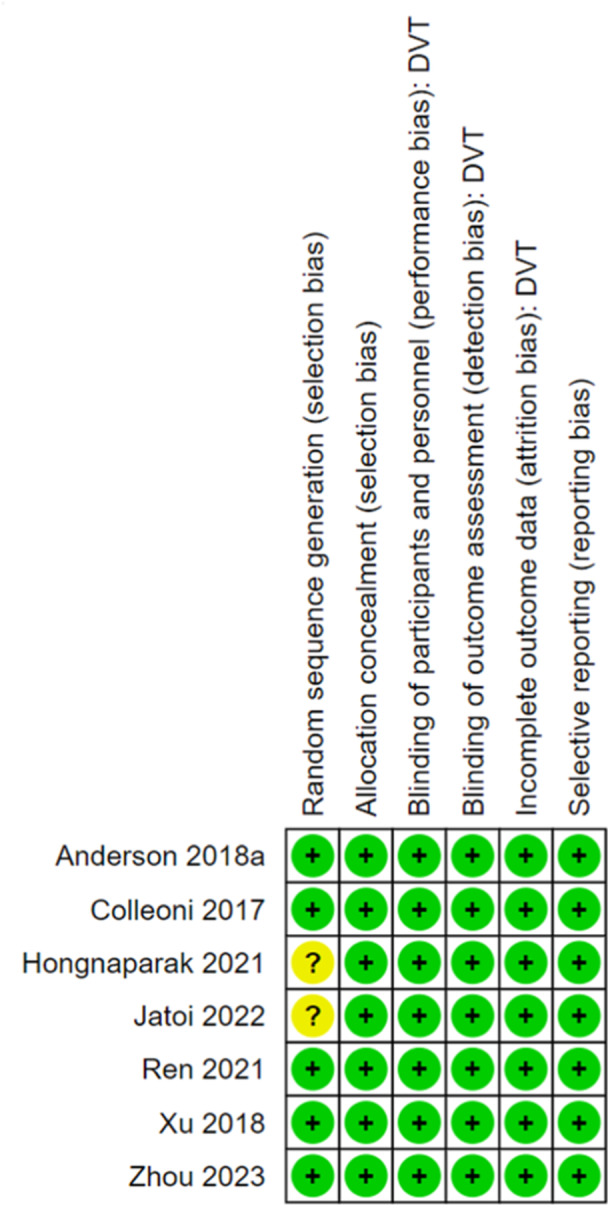
Risk of bias tool for the included studies.

The Cochrane risk of bias tool demonstrated that included studies had relatively low levels of bias. Two studies by Jatoi et al. [15] and Hongnaparak et al. [13] did however have some concerns regarding randomisation bias. The former utilised labelled envelopes that were handed out by the researcher to each participant, whilst the latter did not indicate how participants were randomised, only stating that they were. This is summarised in Figure 5. A funnel plot was produced which showed symmetrical distribution around the combined effect size line indicating a low risk of publication bias. This is found in the supplementary file.

### Primary analysis

**Figure 6:**
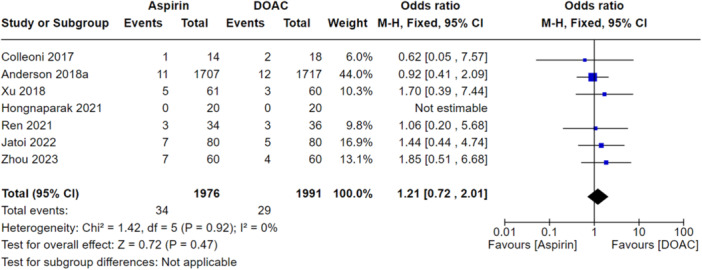
Comparison of all VTEs between the aspirin and DOAC groups.

**Figure 7:**
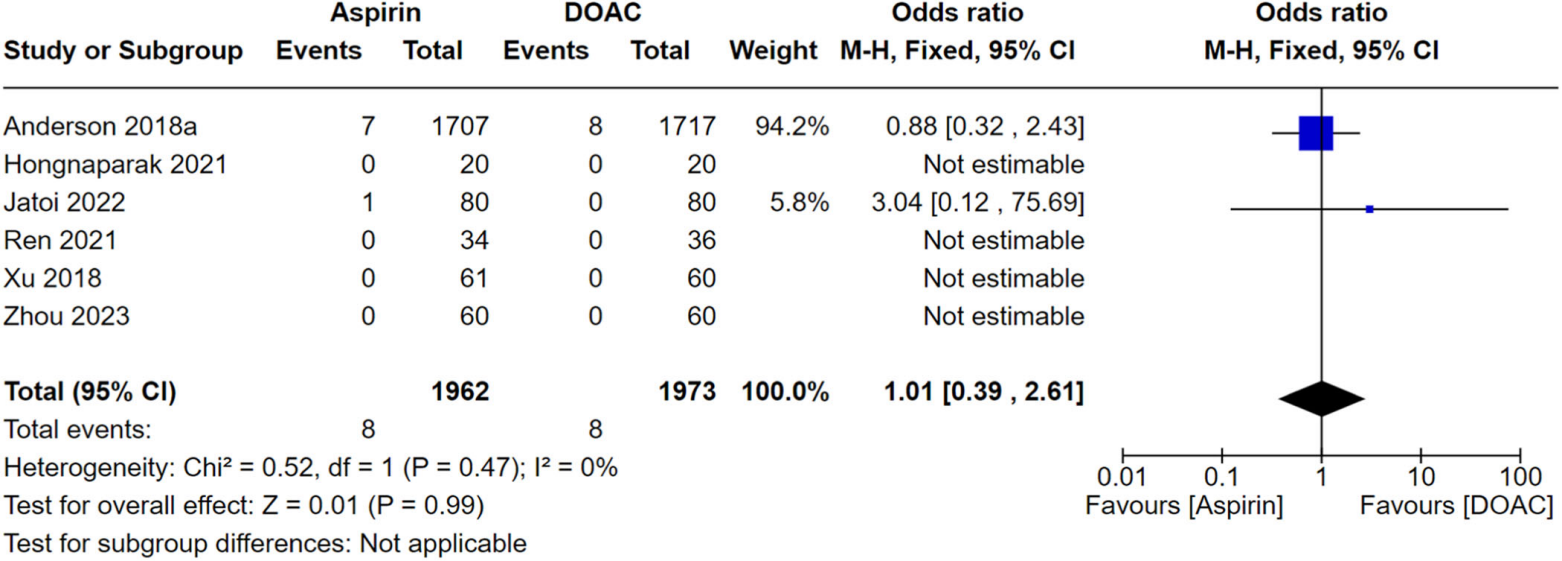
Comparison of the rates of pulmonary embolism between the aspirin and DOAC groups.

**Figure 8:**
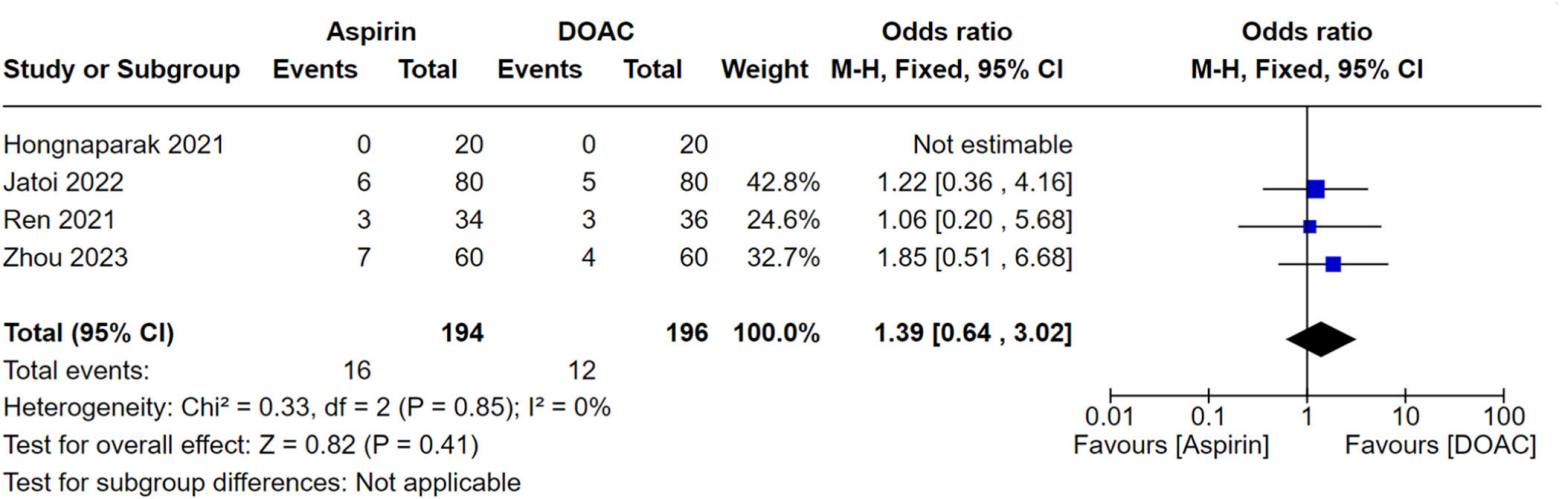
Comparison of the rates of asymptomatic DVTs between the aspirin and DOAC groups.

**Figure 9:**
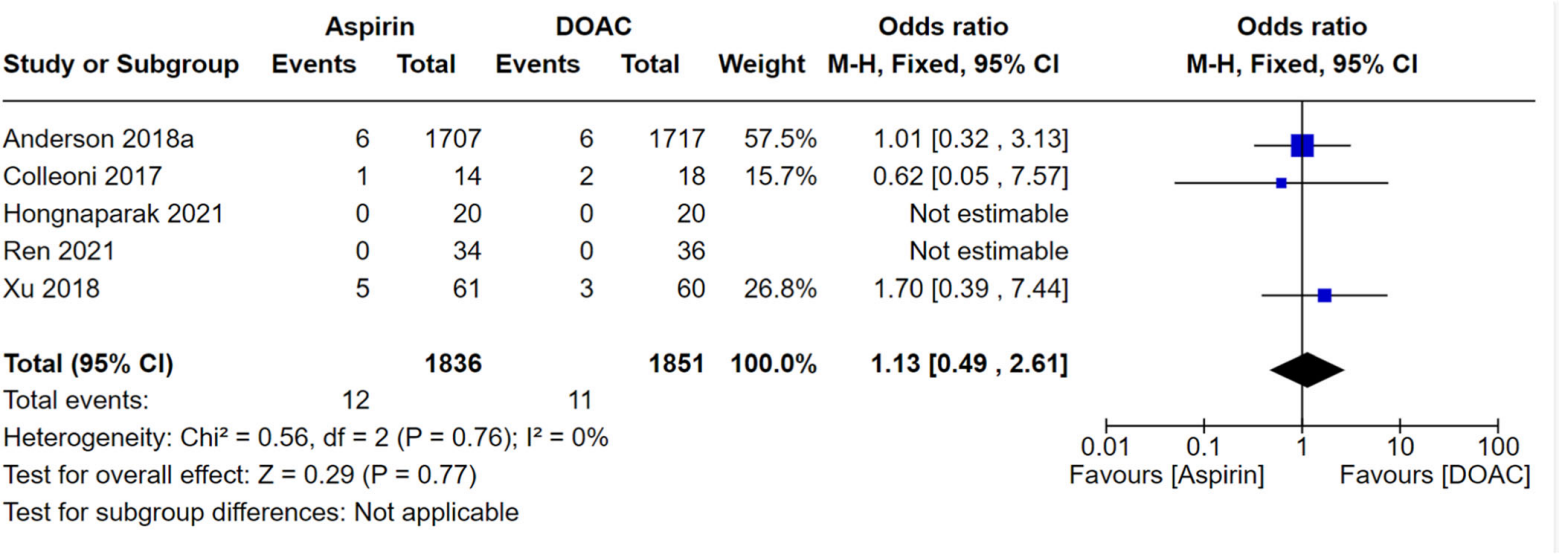
Comparison of the rate of symptomatic DVTs between the aspirin and DOAC groups for the included studies.

When looking at all VTEs (DVT and PE), there was no significant difference between the aspirin and DOAC groups (OR: 1.21, 95% CI: 0.72–2.01), indicating they performed similarly at preventing all VTEs as shown in Figure 6. When this was stratified for PEs only, this was also not significantly different between the groups (OR: 1.01, 95% CI: 0.39–2.61) as shown in Figure 7. There were also no significant differences between aspirin and DOAC groups for asymptomatic DVTs (OR: 1.39, 95% CI: 0.64–3.0) and symptomatic DVTs (OR: 1.13, 95% CI: 0.49–2.61) as shown in Figures 8 and 9, respectively. All the analyses conducted in our primary analysis had acceptable levels of heterogeneity negating the need for subgroup analyses.

### Secondary analysis

**Figure 10:**
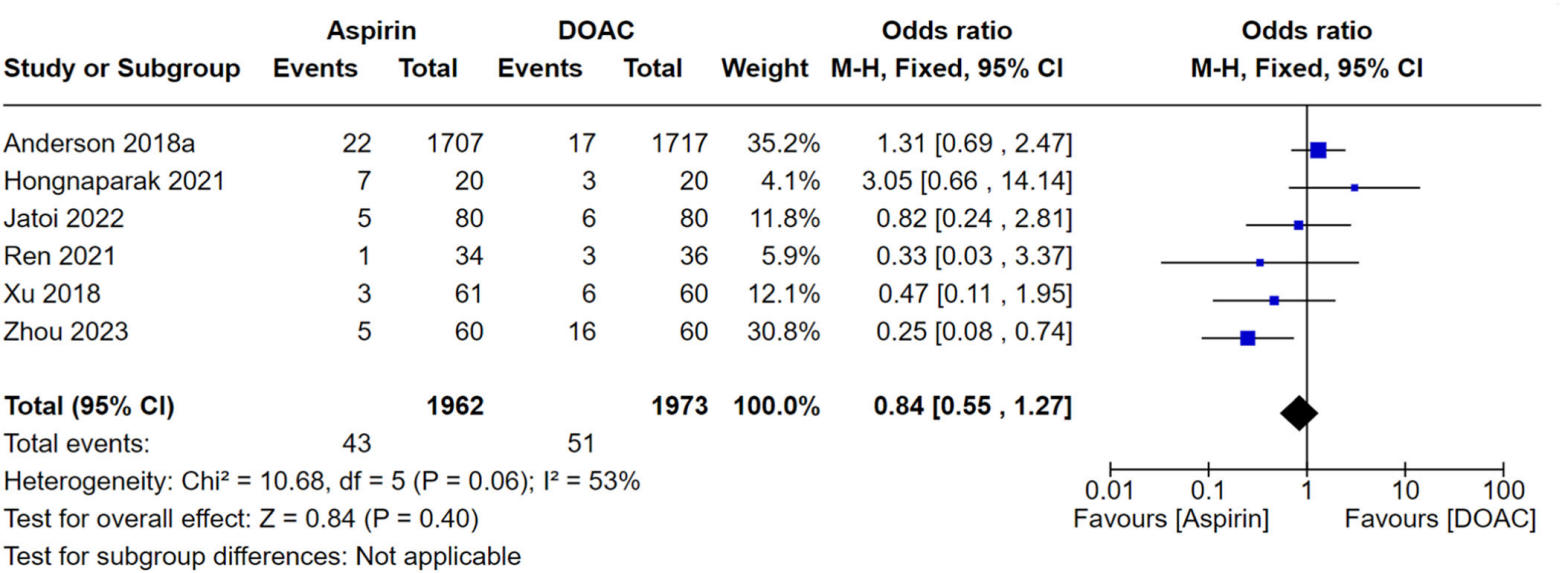
Comparison of the rate of any bleeding event between the aspirin and DOAC groups.

For the secondary analysis, the included studies only reported the rates of bleeding events. Only two studies reported mortality data and major bleeding data; these analyses can be found in the supplementary file. The comparison showed that there was no difference in the rates of bleeding events for the groups (OR: 0.84, 95% CI: 0.55–1.27). The results of this analysis are shown in Figure 10. Low‐to‐moderate levels of heterogeneity were seen in this analysis but were more than likely seen due to some studies having small sample sizes.

## DISCUSSION

Our study has analysed the largest number of RCTs to date looking at aspirin versus DOACs as VTE prophylaxis specifically in the context of elective TKA and THA. Our strict inclusion criteria have strengthened our findings by allowing us to draw conclusions in very specific clinical scenarios. While we initially aimed to include all DOACs in our study, the only DOAC used in included studies was rivaroxaban at a once‐daily dose of 10 mg which allows us to specifically draw a conclusion on this dose of rivaroxaban versus aspirin after TKA and THA VTE prophylaxis.

In our primary analysis, we found no significant difference between rivaroxaban and aspirin for all VTEs. This was also the case for PE, although only two studies reported this outcome. There was also no difference in the rates of asymptomatic and symptomatic DVTs. These results suggest that aspirin is equally effective as rivaroxaban for VTE prophylaxis in patients after primary elective TKA and THA surgery. Additionally, our review found no difference in bleeding events between the two groups, indicating comparable safety profiles. In terms of implications for practise, aspirin as the cheaper, cost‐effective medication, may constitute an additional medication clinicians can use routinely for patients for this specific indication (primary elective TKA and THA surgery) [16].

Our study agrees with the 2020 meta‐analyses of Xu et al., which compared aspirin and rivaroxaban for VTE prophylaxis after TKA or THA, with the inclusion of non‐RCT evidence [32]. They concluded that aspirin was not significantly different than rivaroxaban at preventing VTE with a similar safety profile. However, our study disagrees with the results of Le et al.'s analyses, who concluded that aspirin had equal efficacy at preventing VTE when compared to rivaroxaban, but rivaroxaban was superior in reducing the DVT risk [18]. Given the inclusion of lower classes of evidence, this may explain a difference in conclusions. Despite this, we do believe our respective studies all suggest aspirin's use as a VTE prophylaxis agent is similar to rivaroxaban in terms of clinical outcomes and safety and should be considered a viable alternative to DOACs.

Our study looked specifically at the elective arthroplasty setting. However, when studies considered cases other than TKA or THA for VTE prophylaxis our results were no longer concordant. Hu et al. reported that, with the inclusion of hip fractures, aspirin had significantly less non‐major bleeding, but was inferior to Rivaroxaban for VTE prophylaxis [14]. However, their analysis also included cohort studies. Furthermore, Xie et al. performed their analysis of rivaroxaban versus aspirin not limited to orthopaedic patients and echoed these results, with rivaroxaban having better efficacy but more side effects [31]. This could suggest that aspirin may only be a viable anticoagulant regime for elective TKA and THA, but not for arthroplasty post‐trauma.

While we have demonstrated that aspirin may be as effective as rivaroxaban, what is yet not clear is the optimal dosing regimen for aspirin. This is because various studies use different dosing regimens for varying timeframes (14–90 days). Hence, further studies are needed to describe optimal dosing regimens and whether lower dosages may be as effective for VTE prophylaxis. However, it is worth considering that just over 85% of the 1976 patients who received aspirin as thromboprophylaxis within this meta‐analysis were on a low daily dose of 81 mg. Furthermore, follow‐up should be of adequate duration as VTEs occur at a median time of between 12 and 34 days after THA and TKA [5]. VTEs may also occur up to 6 months post‐surgery and further high‐quality trials with sufficient follow‐up duration are required. Hence, new larger studies with extended follow‐up and more information on optimal dosages would help to answer these questions.

The Pulmonary Embolism Prevention after Hip and Knee Replacement trial could allow us to answer questions regarding the long‐term efficacy and dosing of aspirin [23]. This is set to conclude in July 2024, but implications due to COVID‐19 may cause delays. In the meantime, this review summarises available high‐quality clinical evidence comparing the efficacy of DOAC vs aspirin for VTE prophylaxis after THA and TKA.

According to current NICE guidelines, aspirin is licensed as an off‐label prescription for VTE prophylaxis in limited scenarios post‐arthroplasty [21]. These may include patients who have bleeding disorders or are at a high bleeding risk including those patients who have malignancy [16]. This meta‐analysis supports the use of aspirin in situations where DOACS are contraindicated. Furthermore, should issues regarding dosages and long‐term efficacy be addressed aspirin may also be appropriate in a wider patient population including routine use in the elective primary hip and knee arthroplasty setting.

The analysis of cost‐benefit factors related to aspirin and DOACs was not included in this review, although treatment costs are a significant concern for orthopaedic surgeons and healthcare financiers. Currently, a VTE prophylaxis course with factor Xa inhibitors can be up to 1980.6% more expensive than aspirin [4]. However, as patents for DOACs expire within the next decade, the cost difference is expected to decrease with the introduction of generic DOACs [20]. Future analyses based on these generic versions will be necessary for an effective cost–benefit analysis. Our findings suggest that aspirin is equivalent in terms of efficacy and safety, which may inform these future evaluations.

## CONCLUSION

In conclusion, our systematic review and meta‐analysis have demonstrated that aspirin is as effective as rivaroxaban for preventing VTEs, including PE and DVT with comparable safety profiles for patients undergoing primary elective TKA and THA. The results from this review does support current NICE guidelines recommending aspirin, albeit the recommendation is only for very limited clinical scenarios. However, in the future, a broader use of aspirin as a VTE prophylaxis agent may be considered should several key questions be answered. These include issues regarding the long‐term efficacy of aspirin as well as optimal dosing regimens for patients, with this remaining a novel area of research.

## AUTHOR CONTRIBUTIONS

**Fauzaan Ali Syed**: Writing—original draft; formal analysis; data curation; methodology. **Hamzah Amin**: Writing—original draft; writing—review and editing; formal analysis. **Biju Benjamin**: Writing—review and editing; supervision. **Michiel Hendrix**: Writing—review and editing; supervision. **Terence Savaridas**: conceptualisation; supervision; writing—review and editing.

## CONFLICT OF INTEREST STATEMENT

The authors declare no conflicts of interest.

## ETHICS STATEMENT

The authors have nothing to report.

## Supporting information

Supporting information.

## Data Availability

This study uses previously published data.
