# Supplementary material for: Comparative efficacy of aspirin versus direct oral anticoagulants for venous thromboembolism prophylaxis following primary total hip arthroplasty or total knee arthroplasty: A systematic review and meta‐analysis of randomised controlled trials
Source: J Exp Orthop. 2024 Sep 2;11(3):e70010. doi: 10.1002/jeo2.70010 (PMC11366972; doi:10.1002/jeo2.70010)
Supplement: Supplementary file 1 — Supporting information. [file JEO2-11-e70010-s001.docx]

Supplementary file:

Figure1 : Funnel Plot showcasing publication bias across RCTs utilised.


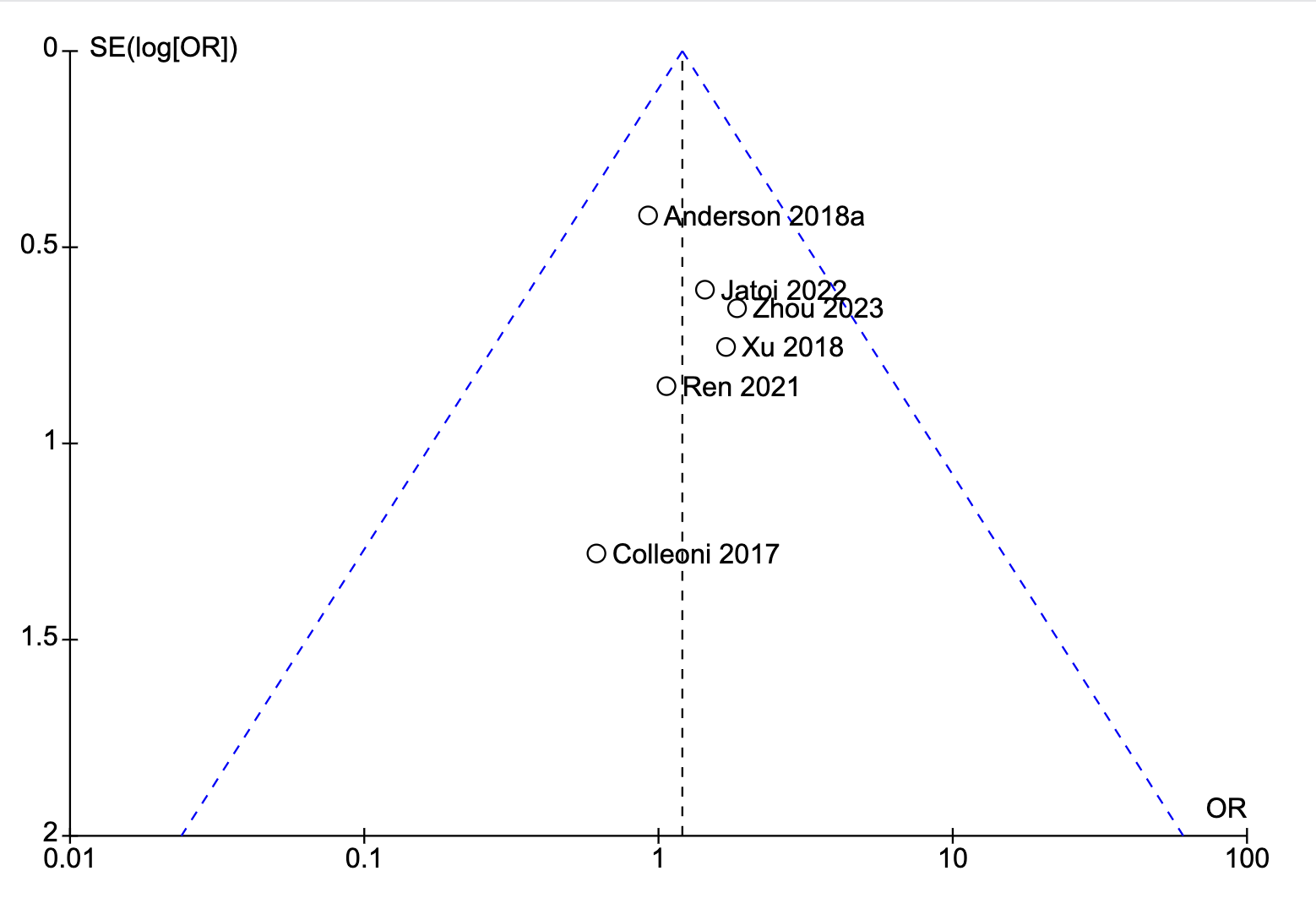


Figure1 : Funnel Plot showcasing publication bias across RCTs utilised.


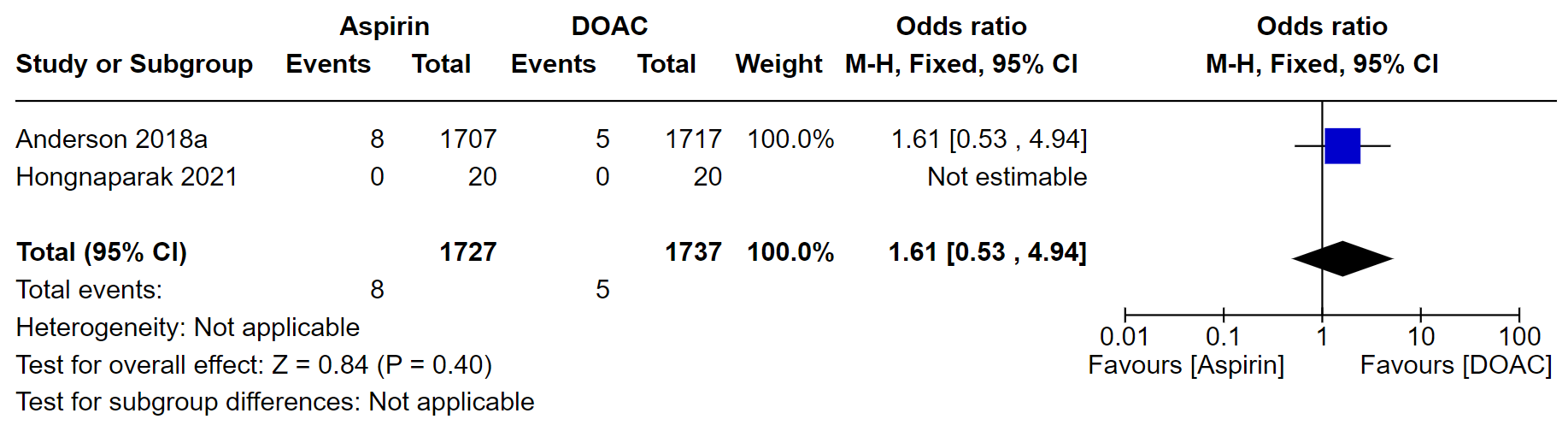


Figure 2: Comparison of Major Bleeding events between the Aspirin and DOAC groups


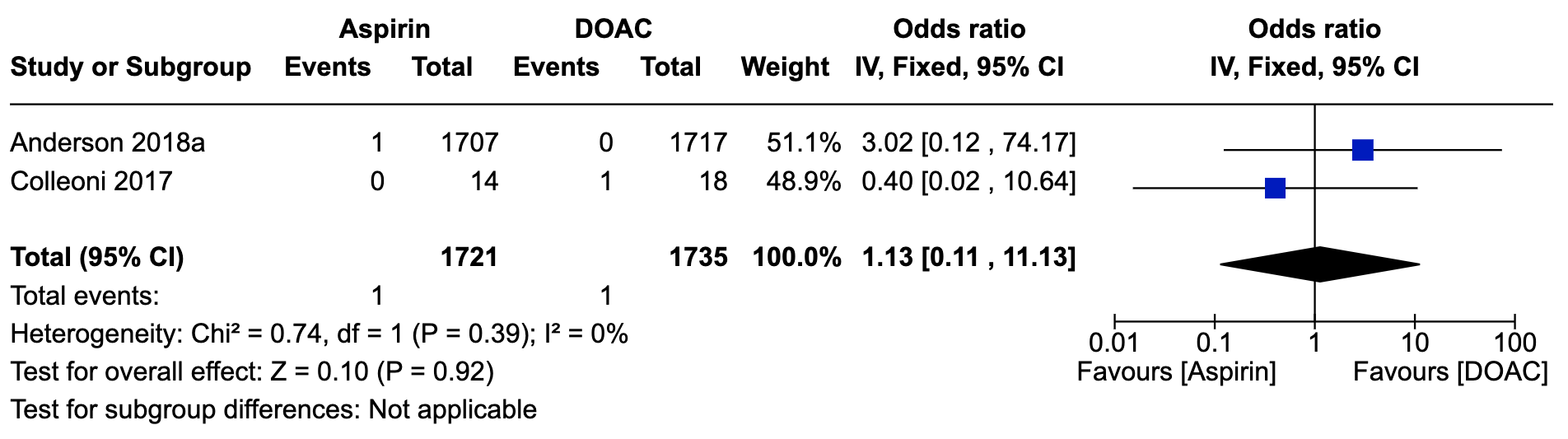


Figure 3: Comparison of Mortality events between the Aspirin and DOAC groups
